# Implementation of evidence-based multiple focus integrated intensified TB screening to end TB (EXIT-TB) package in East Africa: a qualitative study

**DOI:** 10.1186/s12879-023-08069-3

**Published:** 2023-03-14

**Authors:** Kahabi Isangula, Doreen Philbert, Florence Ngari, Tigest Ajeme, Godfather Kimaro, Getnet Yimer, Nicholaus P. Mnyambwa, Winters Muttamba, Irene Najjingo, Aman Wilfred, Johnson Mshiu, Bruce Kirenga, Steve Wandiga, Blandina Theophil Mmbaga, Francis Donard, Douglas Okelloh, Benson Mtesha, Hussen Mohammed, Hadija Semvua, James Ngocho, Sayoki Mfinanga, Esther Ngadaya

**Affiliations:** 1 Muhimbili Centre, National Institute for Medical Research, Dar Es Salaam, Tanzania; 2 School of Nursing and Midwifery, Aga Khan University, Dar Es Salaam, Tanzania; 3 Center for Innovative Drug Development and Therapeutic Trials for Africa (CDT-Africa), Addis Ababa University, Addis Ababa, Ethiopia; 4 Center for Global Genomics & Health Equity, Department of Genetics, Perelman School of Medicine, University of Pennsylvania, Pennsylvania, USA; 5 Alliance for Africa Health and Research (A4A), Dar Es Salaam, Tanzania; 6 Lung Institute, College of Health Sciences, Makerere University, Kampala, Uganda; 7 Division of Infection and Global Health, School of Medicine, University of St Andrews, St. Andrews, UK; 8 Kenya Medical Research Institute, Kisumu, Kenya; 9 Kilimanjaro Clinical Research Institute and Kilimanjaro Christian Medical University College, Moshi, Tanzania; 10 College of Medicine and Health Sciences, Dire Dawa University, Dire Dawa, Ethiopia

**Keywords:** Tuberculosis, Case detection, Screening, EXIT-TB, Tanzania, Uganda, Kenya, Ethiopia

## Abstract

**Introduction:**

Tuberculosis (TB) remains a major cause of morbidity and mortality, especially in sub-Saharan Africa. We qualitatively evaluated the implementation of an Evidence-Based Multiple Focus Integrated Intensified TB Screening package (EXIT-TB) in the East African region, aimed at increasing TB case detection and number of patients receiving care.

**Objective:**

We present the accounts of participants from Tanzania, Kenya, Uganda, and Ethiopia regarding the implementation of EXIT-TB, and suggestions for scaling up.

**Methods:**

A qualitative descriptive design was used to gather insights from purposefully selected healthcare workers, community health workers, and other stakeholders. A total of 27, 13, 14, and 19 in-depth interviews were conducted in Tanzania, Kenya, Uganda, and Ethiopia respectively. Data were transcribed and translated simultaneously and then thematically analysed.

**Results:**

The EXIT-TB project was described to contribute to increased TB case detection, improved detection of Multidrug-resistant TB patients, reduced delays and waiting time for diagnosis, raised the index of TB suspicion, and improved decision-making among HCWs. The attributes of TB case detection were: (i) free X-ray screening services; (ii) integrating TB case-finding activities in other clinics such as Reproductive and Child Health clinics (RCH), and diabetic clinics; (iii), engagement of CHWs, policymakers, and ministry level program managers; (iv) enhanced community awareness and linkage of clients; (v) cooperation between HCWs and CHWs, (vi) improved screening infrastructure, (vii) the adoption of the new simplified screening criteria and (viii) training of implementers. The supply-side challenges encountered ranged from disorganized care, limited space, the COVID-19 pandemic, inadequate human resources, inadequate knowledge and expertise, stock out of supplies, delayed maintenance of equipment, to absence of X-ray and GeneXpert machines in some facilities. The demand side challenges ranged from delayed care seeking, inadequate awareness, negative beliefs, fears towards screening, to financial challenges. Suggestions for scaling up ranged from improving service delivery, access to diagnostic equipment and supplies, and infrastructure, to addressing client fears and stigma.

**Conclusion:**

The EXIT-TB package appears to have contributed towards increasing TB case detection and reducing delays in TB treatment in the study settings. Addressing the challenges identified is needed to maximize the impact of the EXIT-TB intervention.

**Supplementary Information:**

The online version contains supplementary material available at 10.1186/s12879-023-08069-3.

## Introduction

The persistent burden of Tuberculosis (TB) in sub-Saharan Africa (SSA) is linked to missed diagnoses, delayed diagnoses, and challenges with access to high-quality care, which all continue to contribute to a higher risk of death, suffering, and catastrophic financial consequences [1]. While it is well recognized that early TB diagnosis and prompt treatment are cornerstones to TB control, early case detection, and timely treatment also reduce morbidity and mortality associated with TB [2]. TB is a health priority in SSA where lack of modern facilities for proper diagnosis and management has left majority of patients undiagnosed, and these continue to spread the disease. In most SSA countries, TB case finding is through passive case finding and where possible, provider-initiated active case finding of symptomatic people in a predetermined target group such as HIV-infected individuals [1–3]. Under passive case finding, an individual is required to report to a health facility for care. In Tanzania, Kenya, Uganda, and Ethiopia, for an individual to be recognized as a presumptive TB patient, they need to report to a health facility with a cough of two or more weeks with or without accompanying symptoms [4–6], limiting the opportunity to capture those who report less duration cough, women and children attending Reproductive and Child Health (RCH) clinics and diabetic patients. To a large extent, passive case finding depends on individuals’ self-initiative to visit a healthcare facility and report a cough with proper duration, socio-economic status, and knowledge, and on the degree of alertness of health workers to suspect a patient [7].

In the East African (EA) region, TB case detection is below what is required to achieve the World Health Organization (WHO) END TB strategic target of reducing TB incidence by 50% by 2025 [4–6] and is even lower among women and children. The 2021 National TB control (NTP) reports show that TB case notifications per 100,000 population were 134 in Tanzania, 135 in Kenya, 155 in Uganda, and 84 in Ethiopia, and in most countries only 40% were females [2–6]. These values appear to be less than the estimated incidences for 2021 of 208 in Tanzania, 252 in Kenya, 199 in Uganda and 119 in Ethiopia per 100,000 population [2–6]. Moreover, TB case detection at Care and Treatment Centres (CTC) is based on the screening of symptomatic patients and none is done at diabetic clinics. Furthermore, although TB contact tracing is one of the core NTP activities in all four countries [2–6], it is not sufficiently done even among contact children due to financial constraints. It is known that women are good attendants of RCH clinics either for their reproductive health services or the health of their children, therefore, the current practice limits the opportunity of capturing TB cases among women and their children, and hence limits the efforts of NTP’s of putting more infectious TB cases into care. In addition, although chest X-ray (CXR) has been recently promoted and recommended by WHO as a useful tool for TB screening and triaging algorithms [8], none of the NTPs in the region has adopted it into policy. CXR has been reported to be the most sensitive TB screening tool (with very low specificity though) since a significant proportion of TB patients are asymptomatic [9]. CXR when used to triage who should be tested with GeneXpert has been reported to reduce the number of individuals to be tested with the assay, and thus, reduces high costs associated with GeneXpert and thus improve the efficiency of GeneXpert [10].

Given inadequate TB case detection and high mortality especially among HIV patients, RCH clients and diabetic patients, interventions to increase detection, and reduce diagnostic and treatment delays and mortality are needed to contribute towards improving the situation in SSA. Therefore, an Evidence-Based Multiple focus Integrated Intensified TB Screening package (EXIT-TB) was implemented in EA. The EXIT-TB package consisted of integrating TB case detection activities in RCH and diabetic clinics, systematic TB symptoms screening among health facilities attendees using healthcare workers or community health workers (CHW’s), followed by CXR wherever available, screening for TB irrespective of symptoms among HIV infected individuals with advanced disease attending CTCs, targeted contact tracing for all TB patients with a child household member, and use of stool GeneXpert among children who could not expectorate, and a TB diagnostic test (either smear microscope or GeneXpert). This paper explores the accounts of participants from Tanzania, Kenya, Uganda, and Ethiopia regarding the contribution and implementation of the EXIT-TB intervention and suggestions for scaling up.

## Methods

### Design

The parent study was a multi-country cluster randomized controlled trial where the unit of randomization was the facility [11]. The EXIT-TB package was implemented in a total of 4 urban and 3 rural facilities in each participating country. Twenty-eight facilities (7 in each country) were randomized to either early intervention or deferred arm. The intervention was done in the first 12 months in 16 facilities and deferred in 12 facilities (control sites). Implementing the intervention in all 28 clinics simultaneously was a major challenge—hence we opted for this approach to scale up sequentially. While the EXIT-TB package was implemented, a qualitative descriptive approach was envisaged in gathering insights from the participants on EXIT-TB package implementation. This approach was deemed appropriate to answer three key questions to this inquiry: (i) What is the contribution of the EXIT-TB package on TB case detection in the study settings? (ii) what are the challenges encountered and possible solutions that were taken during the implementation of the EXIT-TB package? and, (iii) What are the key considerations for scaling up the EXIT-TB package? A qualitative descriptive approach is appropriate for this inquiry as it aims to develop an understanding and describe the contribution of the EXIT-TB package on TB case detection without testing an existing theory [12]. This approach offered an effective way of gaining a deep and rich understanding of HCWs, CHWs, and stakeholders’ perceptions and experiences in the chosen context, as this may differ from other contexts in terms of culture, expectations, and resources within health care settings.

### Settings

In each country, regions/districts/counties and facilities were selected for the EXIT-TB implementation. Dar es Salaam in Tanzania, Nakaseke and Kiboga in Uganda, Siaya in Kanya Dire Dawa, and Addis Ababa in Ethiopia were selected because they have the highest TB rates and human immunodeficiency virus (HIV) co-infection in the region [3, 13–15]. Furthermore, the EXIT-TB package was implemented in seven healthcare facilities in each country with four facilities commencing implementation early (early implementation sites) as compared to the rest. Therefore, qualitative data were collected in these 4 early implementation facilities in each country because participants had much longer exposure to the intervention.

### EXIT-TB intervention package

The EXIT-TB package involving integrated TB case-finding activities was implemented from April 2019 to January 2022. The package was implemented at the reproductive and child health clinics (RCH), diabetics and HIV clinics in addition to the outpatients’ departments (OPD) using systematic TB symptom screening at these service delivery points [11]. We introduced a stamp of TB symptoms in all patient forms to aid in TB symptom screening. This was followed by further clinical evaluation by the clinicians at the OPD, RCH, and diabetic and HIV clinics. Healthcare workers and/or CHWs at the entry point of the OPD, RCH, diabetic and HIV clinics were trained on how to screen for TB symptoms. Following clinician evaluation, all symptomatic patients defined as patients who either reported a cough and/or haemoptysis of any duration or excessing weight loss, or excessive night sweats or fever were further screened using CXR (with exception of pregnant women, diabetic patients, and HIV infected individuals who were directly subjected to TB diagnostic test). Contact tracing among children with a household member with TB and CXR of all symptomatic children were also performed. Following CXR findings, patients were triaged accordingly, and presumptive TB patients were either tested using smear microscopy or GeneXpert (depending on availability). Presumptive TB patients were grouped as follows (i) Those with CXR suggestive of TB regardless of the presence of other TB cardinal symptoms, (ii) Patients with a short duration cough with CXR suggestive of TB regardless of other TB cardinal symptoms, (iii) Patients with a long duration cough defined as cough of two or more weeks regardless of the CXR findings, (iv) Diabetic patients with cough and/or haemoptysis of any duration, (v) HIV infected individuals (stage 1 and 2) with any of the TB symptoms regardless of the duration of the TB symptoms, (vi) HIV infected individuals with advanced diseases (stage 3 and 4) regardless of the presence of cough, and (vii) Pregnant women with a cough with or without any other TB cardinal symptoms or haemoptysis regardless of duration. To minimize the cost of CXR, the project facilitated the procurement of an X-ray machine in one of the facilities in Kenya and met the cost for presumptive patients in other facilities (in all countries) for patients from low-income families. Only two facilities (one in Kenya and one in Tanzania) had no CXR services. In Kenya, the project paid for CXR services for presumptive patients from low-income families in a facility located about 2 kms from the project site however they were required to meet the transport costs. In Tanzania, the project facilitated the payment of the cost of CXR for presumptive patients from low-income families in a nearby private facility that was within walking distance of the project site. All diagnosed TB patients were treated according to the National TB treatment guidelines.

### Study population, sample size, and sampling method

A total of 73 in-depth interviews (IDI) were conducted with purposefully selected service providers, patients, policymakers, and stakeholders as we wanted to gather perspectives on EXIT-TB from different healthcare facilities and countries. While equal representation is not a primary focus in qualitative studies [11], the level and ownership of facility (public, private & FBO and dispensary, health centre, and hospital) and country were considered during participants’ enrolment. No age preference was made for this qualitative inquiry other than being a service provider who participated in EXIT-TB implementation, a policy marker who is aware of the EXIT-TB package; a patient who received EXIT-TB intervention; OR a stakeholder working on TB control issues in respective countries.

It is important to note that the National TB Programs are the arms of the Ministry of Health charged with the responsibility of the prevention of TB to a point where it is no longer a major public health concern. In Tanzania, the NTLP (https://ntlp.go.tz/) coordinates all TB, TB-HIV and Leprosy control activities TB activities involve prevention, screening, and treatment. In Uganda, NTLP (https://www.health.go.ug/programs/tb-leprosy-control-program/) is responsible for setting policies, planning, training, procurement of supplies and drugs and setting diagnostic and quality standards for central and peripheral laboratories. Similarly in Kenya, NTLP (https://www.nltp.co.ke/) coordinates all services pertaining to TB, Leprosy and Lung diseases from prevention and health promotion, monitoring and evaluation, community engagement, communication, laboratory, care and treatment, policy and planning, monitoring, evaluation and research, supply chain and pharmacovigilance, and administration and finance. Finally, in Ethiopia, NTP (https://www.moh.gov.et/site/initiatives-4-col/Tuberculosis_and_Leprosy) coordinates TB services and finances.

### Data collection tools

Semi-structured IDI guides were developed and translated through a consultative process involving experts from study countries. First, the English versions of the interview guides were translated into Swahili (Tanzania), Luganda (Uganda) and Amharic (Ethiopia), then back-translated to English, and checked for conceptual equivalence. Questions within the interview guide ranged from those examining experiences of participation in the implementation of the EXIT-TB package, the contribution of the package to the facility, challenges encountered during implementation to suggestions for scaling up. Four (4) research assistants with degrees in Social Sciences and Medicine were recruited in each country and trained on the use of interview guides and techniques about this study. The interview guides were pre-tested in purposefully selected settings which were later excluded from the study. After pre-testing, the guides were refined to ensure readiness for use in the actual data collection process. Support supervision of research assistants was conducted throughout the data collection and analysis stages to ensure data quality.

### Recruitment of participants

Participant recruitment was guided by the desire to maximise data source triangulation (a key aspect of qualitative research rigor). We, therefore, sought to include participants who performed various duties during the implementation of the EXIT-TB package. Clinicians provided clinical consultation services for TB presumptive patients and made the diagnosis. Nurses were involved in TB screenings, patient education and treatment supervision. Community health workers and volunteers were involved in initial TB screening and patient enrollment. Together with community health workers, link assistants provided linkage of resumptive patients from the community to health facilities and tracking of lost to follow-ups. Heads of facilities and departments were involved in the coordination of EXIT-TB activities. Finally, TB coordinators and stakeholders at the local and national levels provided technical support and coordination of EXIT-TB activities at their respective levels. Therefore, the recruitment for service providers commenced with a purposeful selection of healthcare facilities for implementation of the EXIT-TB package in respective countries. Before IDIs, a courtesy visit was made to appropriate local authorities for approval to visit the facility. This was followed by a physical introductory visit to the facility where the study information was provided to the incharge, and subsequently, project implementation personnel were identified. This was followed by subsequent visits by research assistants to schedule and conduct IDIs. Recruitment for EXIT-TB patients were conducted through CHWs and service providers. Recruitment for policymakers and stakeholders was done by initial phone call after obtaining phone numbers from the Regional/county and district/sub-county authorities. During phone calls with policymakers and stakeholders, interviews were scheduled considering participant preferences of place, date and time.

### Conducting interviews

Qualitative interviews were conducted between May and July 2022, three months after the completion of the EXIT-TB package implementation. Interviews were conducted in a place and date preferred by the participants. Before the commencement of IDI, participants were given information about the study, risks, and benefits of participation (an information sheet was part of the interview). Verbal consent for the interview and voice recording was sought in advance and recorded as part of the interview. Then, interview sessions lasted for approximately 30–60 min in a safe environment. The data collection stopped when data saturation was achieved. Because of the COVID-19 pandemic, all participants and research assistants were provided with face masks and hand sanitizers. Social distance was maintained throughout the interviews.

### Data management and analysis

IDI data transcription and translation was done simultaneously by research assistants, and transcripts were verified by the research team in respective countries. Interview transcripts were deidentified, pseudonyms were generated for each participant, and the data was uploaded into the NVivo 12 software (QSR International) for management and deductive thematic coding. A stepwise approach was used for the thematic analysis of the interview transcripts [16]. First, the research team examined the research questions and generated several themes based on consensus. This resulted in an analytical matrix of the main themes and subthemes. Individual transcripts and phrases (codes) representing participants’ responses to investigators’ questions were exported to relevant themes and related sub-themes within NVivo. A consensus-based approach was then used by the research team to decide on including codes that do not fit within the pre-developed sub-themes and themes; the codes were excluded when they did not provide critical value to the study, as confirmed by subjective and objective evaluations. The coded data within NVivo were then exported to Microsoft Word (Microsoft Corporation) for interpretative analysis and report generation.

## Results

### Participants demographic characteristics

The demographic characteristics of participants are represented in and Table 1. In total, 73 participants aged 22–70 years were included in this qualitative audit, with most participants aged between 41 and 50 years. Most participants 43 (58.9%) were male. Participants included clinicians 24 (32.9%), TB coordinators and stakeholders 17 (23.3%), community health workers 14 (19.1%), medical In-charges 10 (13.7%) and nurses 8 (11.0%).

**Table 1:** Participants’ demographics

	Tanzania n (%)	Kenya n (%)	Uganda n (%)	Ethiopia n (%)	Total
Age					
21–30	1 (3.7)	4 (30.8)	1 (7.1)	3 (15.8)	9 (12.3)
31–40	5 (18.5)	7 (53.8)	7 (50)	6 (31.6)	25 (34.2)
41–50	14 (51.9)	1 (7.7)	5 (35.7)	7 (36.8)	27 (37.0)
50 +	7 (25.9)	1 (7.7)	1 (7.1)	3 (15.8)	12 (16.4)
Sex					
Male	12 (44.4)	8 (61.5)	12 (85.7)	11 (57.9)	43 (58.9)
Female	15 (55.6)	5 (38.5)	2 (14.3)	8 (42.1)	30 (41.1)
Cadres					
Clinician	5 (18.5)	6 (46.2)	5 (35.7)	8 (42.1)	24 (32.9)
CHWs/CHVs	7 (25.9)	3 (23.1)	4 (28.6)	0 (0)	14 (19.1)
Medical Officer in charge	7 (25.9)	1 (7.7)	1 (7.1)	1 (5.3)	10 (13.7)
TB coordinators/Stakeholders	7 (25.9)	3 (23.7)	4 (28.6)	3 (15.8)	17 (23.3)
Nurse	1 (3.7)	0 (0)	0 (0)	7 (36.8)	8 (11.0)
Total	27	13	14	19	73

### Theme 1: The contribution of the EXIT-TB package to TB case detection

#### Increased TB case detection

There was a consensus among most participants that the implementation of the EXIT-TB project has increased TB case detection. The descriptions during qualitative interviews strongly reflected project monitoring and evaluation data in the 16 selected facilities that indicated an increase in TB case detection from 320 in Tanzania, 253 in Kenya, 169 in Uganda and 39 in Ethiopia per 100,000 population during the 2017 baseline survey to 505 in Tanzania, 334 in Kenya, 208 in Uganda and 78 in Ethiopia per 100,000 population at the end of the EXIT-TB project in 2022.The contributors of the EXIT-TB package to increased TB case detection described by participants were six-fold (Table 2). The first contributor was the use of CXR services to screen for TB after symptom screening. In some countries, CXR services were made free, especially for those who couldn’t afford them. Free CXR especially among those who couldn’t afford the cost recurrently emerged as a key driver of increased uptake of TB screening. One participant in Uganda mentioned free CXR services worked better because most people could not afford the cost of X-ray services before the project. Free CXR were described as facilitating access of poor people to this essential service in the TB diagnosis process. Relatedly, there were affirmations in Tanzania and Ethiopia that the project improved access to TB services among the poor and low-income population. In Tanzania, some participants described a tendency of peer referral for free CXR services, particularly in areas with low-income populations. In Ethiopia, the project was cited to have facilitated the treatment of poor communities who could otherwise fail to meet the cost of diagnosis and medical care.

**Table 2:** The contribution of the EXIT-TB Package

Contribution of EXIT-TB package	Specific contributors	Illustrative quotes
Increased TB case detection	Use of X-Ray services	*The main contribution of EXIT-TB was improved case identification. Before the project, the facility was identifying less than 50 cases in a year. But when EXIT- TB came, for the first time we were able to find more than 100 cases in one year. So active systematic TB symptoms screening improved case identification, especially within the EXIT-TB project. We were able to diagnose more patients through the use of X-rays as both a screening and diagnostic tool. This is because EXIT-TB was supporting free X-Ray services, especially among those who couldn’t afford them. [The project] was paying for the X-rays. So, you're able to diagnose more TB cases, even using X-rays, something that we were not able to do before EXIT-TB (Clinician, Uganda)*
	Engagement of CHWs	*Working with [CHWs] facilitated the smooth implementation of the EXIT- TB programme by helping patients who were in the programme to get the right services at the right time and reduce costs. It was cost-effective for the patients. They received the right services from the time they entered the facility beginning with health education provided to them to increase their confidence in the TB screening process…those who were found to have TB were immediately put on medications and followed up (Clinical Officer, Tanzania)*
	Increased community awareness and linkage	*One of our responsibilities was to make the community members know and understand the existence of TB in the community. Awareness raising was effective in the catchment area because if someone goes to the community today and asks questions about TB people will be able to answer. We did not only teach them about the existence of the disease but also how the disease is transmitted, how to prevent it and where to go for treatment. We conducted symptoms screening and referred symptomatic patients for further investigation. In the course of the investigation, we were able to find patients who are TB positive and directed them to treatment (CHW, Uganda)*
	HCWs support to CHWs	*Healthcare workers provided support and guidance to us. We were free to move to different departments. Providers prioritised us and gave us an opportunity to talk to patients and we were given a priority to see clinicians (CHWs, Kenya)*
	Screening infrastructure	*We used to do TB Screening and triage at OPD, but it was just an open space. Then when EXIT-TB came in, we realised that we need more space with partitions for specific tasks. We, therefore, created three partitions, one for screening, one for coughing patients and another one for registering new patients, and then we have the other one for HIV screening (Nursing Officer, Uganda)*
	New screening criteria and training	*EXIT-TB came up with a different approach from the existing national guideline. For example, the national guideline considered coughs of fourteen days or more as a proxy for TB screening. However, with the exit TB project implementation, this practice was changed and anyone with a cough of any duration was screened. This new practice facilitated early detection. The screening was also mandatory for newly diagnosed HIV patients, those in stage 3 and 4 patients. Patients with cough of any duration were screened and sent to clinicians for further evaluation and diagnosis. Whenever a patient presented with any TB symptom it was a must to be screened and this was very decisive and facilitated early detection and reduced patient suffering. Previously, patients were referred to the laboratory for diagnosis at a very late stage…after developing the disease and after they became very ill. However, this project accepted patients even those without a single symptom especially those with HIV stage 3 or stage 4… they were all screened and diagnosed (TB Focal Person, Ethiopia)*
Reduced delays in TB Care	Speedful screening, sample collection and processing, and results provision	*One of the good things we started was giving priority to patients with coughs. Previously they were treated with other patients. The other thing is that there was no cough clinic at the beginning, but it was established during the time of the EXIT TB project and enabled us to isolate coughing patients from other clients. Isolated clients got early treatment (ART Head, Ethiopia)* *Whenever we went to the hospital, we were screened quickly for TB infection, sent to the doctor and samples taken quickly. If the result is negative, we were told about the precautions that we must take and in case someone tests positive, they put to start the medications right away (EXIT-TB patient, Uganda)*
Improved capacity and decision-making among HCWs	Training, co-learning and peer mentorship	*Through this study, we got more knowledge, and we met our TB targets because during that time TB was a problem. People did not have adequate knowledge about TB and especially in the entire area and even within the hospital. some did not have enough knowledge… even our staff within the hospital didn’t have enough knowledge about TB. Most of these staff got experience as they were implementing Exit-TB, and we used to have CMEs which further strengthened their knowledge of TB, how it is transmitted and how to screen. We were able to sensitise them, give them knowledge in their different departments, and mentored and supervised them. Before the project, we were getting a small number of TB clients but when EXIT- TB came, we were able to increase the number of TB clients… (TB Clinic Focal Person, Uganda)*
Reduced Lost to follow-up clients	Strong contact management systems, referral system, engagement of TB focal persons and link assistants	*We were able to increase our TB case identification by around 30% or more because of the contact management that we put in place. We were able to identify other cases from the community, especially children and refer them to the facility. That is another one. Also, we were able to minimise the issues of lost clients because once the client comes into the system, we were able to closely follow up through the engagement of the TB focal person. If the client has not reported to the facility, then we will report to TB focal person and link assistants who conducted defaulter management activities. I think we only lost one case throughout the project compared to previous years where we would lose up to four cases. The lost client was an extreme case because a client committed a legal offence and ran away. We exhausted all efforts to contact him unsuccessfully. Reduced lost clients increased our treatment success rates (Clinical Officer, Kenya)*

The second contributor was the engagement of community health workers (CHWs) to offer health education and perform screening at different service delivery points within the facility. CHWs emerged as the dominant group of providers who facilitated a range of TB-related services. Most CHWs affirmed conducting ‘health talks’ to prepare patients to undertake to screen. They also conducted screening at different service points, assisted presumptive patients to be reviewed by doctors, and assisted with access to laboratory and other needed services within facilities. One CHW in Uganda described health talk topics as part of the EXIT-TB package and these topics included: what TB is, what the symptoms are, how TB can be prevented, what to do if they have the symptoms and how they get infected. CHWs in Uganda described receiving patients at the emergency department and facilitating screening after admission into wards. CHWs in Kenya described being involved in contact tracing and client follow-up. The engagement of CHWs in screening was further considered to facilitate patients to receive the right services on time and reduced cost on the part of patients through timely diagnosis and treatment initiation and follow-up.

The third contributor was the increased community awareness about TB as well as the increased linkage of clients, especially children from the community to the facilities. CHWs were described to have been engaged in raising community awareness of TB. Awareness rising included teaching community members about TB transmission, prevention, and treatment. Some CHWs indicated conducting both screening and testing as well as linkage of symptomatic clients to facilities for further management. The linkage of clients from communities to healthcare facilities especially children with a positive TB contact emerged as an important role of CHWs. A CHW in Kenya mentioned having a ‘referral booklet’ that documented presumptive TB patients in the community and referred them to nearby health facilities.

The fourth contributor was the tendency of HCWs to provide support and guidance to CHWs, for instance delegating them to talk to patients, allowing them to move around the facility and consult clinicians at any time, and giving them priority at different departments including laboratory, and offering them a good working environment. The fifth contributor to increased screening and TB case detection was improved screening infrastructure. When asked about the changes EXIT-TB brought to the facility, some participants cited improvements in infrastructure for TB screening. The creation of dedicated space for screening and triage of patients, sample collection and registration were mentioned to have occurred within some facilities as compared to before when all these activities were conducted in one area.

The sixth contributor to increased screening and diagnosis of new TB cases was the adoption of the ‘new screening criteria’ and training of implementors. Issues related to training as part of the EXIT-TB package are detailed below. Participants indicated that the EXIT-TB project embraced new screening criteria that were different from what is described in the existing guidelines. In all countries, for instance, TB suspects as per existing guidelines, are people with a cough for more than two weeks. However, with the EXIT-TB package, anyone with a cough was screened regardless of the duration. Further, EXIT-TB made it mandatory for diabetic patients and HIV-positive patients in stages 3 and 4 regardless of symptoms. Integrating TB case-finding activities in all RCH clinics also contributed to better EXIT-TB performance. The application of these new screening criteria was linked to increased early detection of TB patients and facilitated timely treatment as compared to a previous practice where patients were delayed being presumed due to the long-duration cough criteria. Some participants went further to link the EXIT-TB project with increased detection of Multidrug-Resistant (MDR) TB because of increased use of GeneXpert. For example, an official of at the Kenya NTLP affirmed that

*We were able to diagnose three MDR patients during Exit-TB implementation by using GeneXpert. You know, diagnosing one MDR case is considered the big thing because that is multidrug resistant TB. The patients came through the outpatient department and if we hadn’t placed screeners there, we could have missed them. We were able to diagnose two MDR cases who are HIV negative. This means if we only relied on HIV department, we could have missed them. This was a big deal for us (National level TB personnel, Kenya)*

#### Reduced delays in TB care

Although concerns about, machinery problems, expertise deficits and workload dominated some of the discussion (see below), a few participants affirmed reduced delays and waiting time in TB diagnosis. Speedful screening, speedful sample collection and processing and speedful results provision emerged as drivers of reduced delays. However, this was described as possible in areas where there were no machinery, expertise, or workload challenges. Reduced delays were also cited to result from prioritisation of TB clients and the establishment of special clinics or isolation units for clients with cough who then received much faster care as compared to previous practices where they were treated together with other clients.

#### Improved capacity and decision-making among HCWs

Although few participants in Tanzania affirmed noticing no difference in terms of capacity to implement TB activities between EXIT-TB and routine TB Care, there was a broad consensus among the majority that the project has increased the capacity of HCWs to conduct TB screening and diagnosis, improved decision making among providers and reduced nosocomial infections transmission between providers and clients. For example, a Nursing Officer in Ethiopia mentioned that *the project gave HCWs confidence and courage to conduct a TB risk assessment of clients and make decisions on isolation and treatment and, reduced transmission of respiratory infections from patients to HCPs and *vice versa. Increased capacity among HCWs was linked to increased knowledge and skills acquired through training, co-learning and peer mentorship provided during the implementation of TB activities as part of the EXIT-TB project. For example, a TB Focal person in Ethiopia affirmed working together with other EXIT TB professionals in different roles which facilitated co-learning and increased work effectiveness. There were also affirmations that the project increased knowledge among community members on TB issues that contributed to increased TB diagnoses.

#### Reduced number of lost to follow up clients

Amidst concerns of some clients not returning for sputum testing or treatment (see below), some participants mentioned that EXIT-TB reduced loss to follow-up rates because of strong contact management systems, strong community-facility referral system, engagement of TB focal persons and link assistants who implemented defaulter management activities. The reduced number of clients lost to follow-up was linked to increased treatment success rates.

### Theme 2: Challenges faced in delivering EXIT-TB package

Although few participants affirmed encountering no challenges, most participants mentioned several challenges and barriers. The challenges and barriers encountered during the implementation of EXIT-TB can be heuristically categorised into two groups: *supply-side challenges* and *demand-side challenges* (Table 3). These barriers appeared common in all implementation countries. However, the supply-side challenges dominated compared to the demand-side challenges.

**Table 3:** Challenges faced during the implementation of EXIT-TB Package

Challenges	Specific issues	Illustrative Quotes
Supply side challenges	Service delivery barriers • Disorganized services • Delayed consultations • Short consultation time • Missed screening • Missed investigations • Poor screening integration in routine care • Limited space for TB services • COVID-19 pandemic	*The barriers were so many, but the main barrier was limited contact tracing after identifying patients and starting them on treatment. Since we had small human resources, very few contacts tracing was done, and some clients were not followed up very well (Clinician, Kenya)* *The challenge emerged when COVID-19 started because we were told to close our facility for three months. After all, this was a dedicated COVID-19 care centre. Patients were shifted to another hospital (name) therefore we did not have time to serve them well (TB Focal Person, Tanzania)* *The challenge was COVID-19. You see signs suggesting TB but when you look at the patient and ask some questions about TB there are no symptoms of TB but when you do the COVID-19 test it comes out positive. So, you need to go through both COVID-19 and TB registers (Clinical Officer, Uganda)*
	Human resource barriers • Negativity towards the project due to unmet financial expectations • Fear of TB infection among HCWs • Inadequate staffing and workload • Inadequate knowledge • Fear among providers during COVID-19 • Inadequate financial motivation/incentives to HCWs • Technical barriers Inadequate expertise among some screeners Inadequate expertise in operating some TB diagnostic equipment (e.g., GeneXpert machines) Inadequate expertise in collecting sputum samples from children Inadequate expertise for interpretation of CXR and absence of radiologists in some facilities	*The health workers in (name) Hospital especially the clinicians at the OPD did not welcome the (EXIT-TB) package at the beginning. They were arguing that young children of 7 years and below are not allowed to undergo X-rays as they may face negative health consequences and they were telling us to get sputum samples instead. Nevertheless, those who were unable to provide samples were required to undergo chest X-Ray, but clinicians insisted that it will hurt them if they are aged between 5 and 9 years. This created a disagreement for some time but later the EXIT-TB focal person and District TB focal person enlightened them on the purpose of the study, and that chest is also used routinely to diagnose diseases even among young children, and later started showing cooperation by accepting children for X-Ray services (CHW, Uganda)* *The challenge was a negative attitude among providers towards TB disease; I don't know why. But the attitude of healthcare workers towards TB disease is so negative. Not many HCWs are interested in offering TB care.… healthcare workers sometimes neglect TB cases (because of fear of infection) and this also makes it difficult to treat them (Stakeholder, Uganda)* *What I can say is the people who were helping us in the identification of clients lacked the technical skills. Therefore, we needed frequent mentorship to improve their knowledge and smoothen the implementation of the program (OPD Incharge, Uganda)* *The unavailability of radiologists for urgent cases was a challenge. As a physician, I could go to the X-Ray room and read the results correlating with patients’ symptoms. But other health professionals-health officers and Nurses, might not be able to read the result. Thus, the availability of full-time radiologists or training other cadres was very important (Physician, Ethiopia)*
	Equipment and supply barriers • Inadequate supply of stool containers particularly in Tanzania; • Absence of X-ray machines or dysfunctional X-ray machines • Stock-out of X-ray films and cartilages for GeneXpert machines	*Equipment and supplies were troubling sometimes…., Sputum containers for children were inadequate. X-ray films were another barrier because we did not have enough expertise to read X-rays and we had to go where there is a screen (TB Focal person, Tanzania)* *For children under five years of age, we were taking stool samples for testing in the laboratory and if the results come out positive, we initiated treatment but if negative, s/he was counselled by the doctors. We screened young children by asking questions to their parents. We hand them with stool containers … but sometimes we run out of containers (CHW, Tanzania)* *There were times when many patients were screened and sent for X-rays and GeneXpert, however, the radiologist couldn’t interpret the results from X-rays and therefore could not give the results on time…. There was also a shortage of supplies, shortage of x-ray films, malfunctioning of GeneXpert, cartilage shortage, X-ray machine failure and service interruption. Most of these challenges were because of machine failures (TB Focal Person, Ethiopia)*
Demand side challenges	Negative clients’ behaviours • Delayed care seeking • Late clinic attendance • Coming on non-clinic days • Defaulting care	*The challenge was that patients had less understanding of the disease which necessitated health education. Once you give education and direct them to screening points, some of them refuse because their understanding is poor. They thought that going for screening is an indication that they have TB therefore they started self-stigmatisation but after intensive education, this improved a little bit (TB Focal Person, Tanzania)* *A major challenge within the community was a lack of awareness about the disease. They correlated any weight loss with HIV infection. Also, some of us believe that TB might expose us to stigma and discrimination (EXIT-TB patient, Ethiopia)* *[Patients] fear (to screen) because of fear of stigmatization because they have TB symptoms. You find people fear saying that maybe they have a cough. But we used health education sessions to minimise the fears. People with TB symptoms were separated and seen quicker to reduce the rate of maybe transmission if they have TB” (Clinical Officer, Kenya)* *[Patients] fear (to screen) because of fear of stigmatisation because they have TB symptoms. You find people afraid to say that maybe they have a cough. But we used health education sessions to minimise the fears. People with TB symptoms were separated and seen quicker to reduce the rate of maybe transmission if they have TB” (Clinical Officer, Kenya)* *Another challenge is when you are told to go do an X-ray in a different facility because there are no such services in the screening facility. Some of us could not go to the referral facility because of high transport costs (EXIT-TB patient, Uganda)*
	Limited community awareness and negative beliefs and fears • Poor knowledge of TB diagnosis, treatment, and prevention • Equating screening to having a TB infection • Fear of coming to the facility • Fear of stigma	
	Financial barriers • Failure to meet the cost of care • Failure to meet the cost of transport	

### Supply-side challenges

Participants’ description of supply-side challenges can be grouped into (i) those related to service delivery; (ii) those related to human resource issues and (iii) those related to diagnostic equipment, and supplies. Each of these challenges are examined next.

#### Service delivery barriers

Service delivery barriers included disorganised patient flow across points of care which resulted in unnecessary delays in screening; delays in medical consultation and short consultation time for TB presumptive patients because of high clinicians’ workload; clinicians not performing screening when not done at the entry points, clinicians not performing thorough investigations for TB presumptive patients, poor integration of TB screening in routine clinical investigations and limited space for offering EXIT-TB services within HIV clinics. A key service delivery concern was the COVID-19 pandemic. The challenges introduced by the COVID-19 pandemic were the closure of some facilities particularly those identified as COVID-19 centres, increased demand for personal protective equipment (PPEs) including facemasks which created deficits, fear of some clients attending facilities because of fear of COVID-19 infection (see below) and fear among providers to offer care to patients presenting with cough as they may be dealing with COVID-19 which increased chances of infection. Another challenge was the need to perform TB screening alongside COVID-19 screening which added more documentation responsibilities on the part of the provider. Some of these issues are highlighted in Table 3 below.

#### Human resource barriers

Most participants cited a range of human resource challenges. Human resource barriers included negativity among some clinicians towards the EXIT-TB package at the beginning particularly in Ethiopia and Uganda, although participants in Tanzania and Kenya indicated good reception of the project among HCWs. Negativity towards the project, for instance in Uganda was linked to unmet expectations of financial gain from ‘the research project’, fear of being infected with TB even if they were taught how to prevent themselves from TB infection by the project and fear of negative consequences of CXR among young children. A TB focal person in Uganda attributed the negative attitudes towards the project among providers to the expectation of making money from the project on the part of the staff, which made it difficult to work with them when such expectations were unmet. Likewise, a clinician in Uganda indicated that some HCWs were fearful of working in TB clinics because of fear of infection and preferred to remain in the wards. Relatedly, a volunteer in one hospital in Uganda mentioned that clinicians were hesitant to request for CXR for children under the age of 7 years because of fear that it may contribute to negative health consequences in future. On the contrary, the negativity of HCWs towards the project in Ethiopia was linked to a poor understanding of the project. For example, a TB focal person in Ethiopia indicated that some providers had negative attitudes towards the project at the beginning because they did not have a good understanding of it, and they thought that it only sought to benefit specific people. However, sensitization by the EXIT-TB team was used to address these negativities. Other challenges included inadequate staffing for TB screening; inadequate knowledge among some staff about TB screening, and fear among some CHWs to perform screening and other TB-related activities during the COVID-19 pandemic. Inadequate staffing was cited to contribute to some patients not being screened particularly during weekends when only one clinician is available to deal with patients with all kinds of medical conditions. Understaffing and workload also contributed to poor documentation even among volunteers because of multiple documentation demands and being overwhelmed by clients.

When asked about the level of motivation and the support obtained from leadership, some participants described challenges of reduced motivation and poor coordination at the beginning of the project. Lack of motivation was linked to a lack of financial incentives and high workload. Gaps in coordination at the commencement of the project were linked to a feeling that the project is an added responsibility among providers than routine work partly because of being overburdened by the increased number of clients with no accompanying salary top-ups. This explains why there were concerns about non-payment of financial incentives among some participants in Ethiopia and Tanzania despite working tirelessly on the project. Affirmations of considering the project as additional responsibility and workload were common among participants in Ethiopia as compared to other countries. One participant commented:

*One of the causes for the lack of motivation among the staff was patient load and imbalance between the number of patients attending and the number of staff giving the service. The other thing is that this project work was considered an additional responsibility, so some groups of staff needed additional financial incentives on top of their salary to do this job (OPD Head, Ethiopia)*

There were disagreements among participants on the technical expertise barriers. One group of participants from all countries affirmed facing no technical barriers because of the training provided as part of EXIT-TB implementation. Training on screening among CHWs and training among clinicians on the interpretation of CXR was mentioned regularly by many participants across all countries. Phrases such as ‘there was no problem’, ‘we did not face technical troubles’, ‘we were trained very well’ and ‘we did not encounter challenges’ dominated among many participants. A District TB and Leprosy Coordinator (DTLC) in Tanzania linked the absence of technical challenges to the training of providers on ‘TB and GeneXpert, and that there was a tendency to refer clients for CXR to the nearest facility if the focal facility had no such service and expertise. On the contrary, one group of participants specifically cited technical challenges such as inadequate expertise among some screeners, inadequate expertise in operating some TB diagnostic equipment (e.g., GeneXpert machines); inadequate expertise in collecting sputum samples from children; inadequate expertise for interpretation of CXR and absence of radiologists in some facilities. A clinical officer in Kenya affirmed that “some clinicians did not have expertise in diagnosing TB through CXR”. Similar affirmations of difficulty in CXR interpretation were noted among a few clinicians in Uganda. One participant in Uganda indicated that people who were trained in CXR interpretation were not directly working on CXR-related activities. One participant in Ethiopia indicated that there was an insufficient number of people who could interpret CXR which contributed to delayed results to patients with some waiting for days. This explains why some participants felt that additional training or training of other cadres of providers on CXR interpretation should have been offered during project implementation. It is important to note that concerns about the absence of radiologists were more common in Ethiopia which necessitated using Compact Disks (CDs) to send results to other facilities. This practice was cited to delay results and treatment initiation and increased the chances of disease transmission if the results are suggestive of TB.

#### Equipment and medical supply barriers

There were some disagreements on barriers related to diagnostic equipment and supplies. While most participants in all countries mentioned encountering many equipment and supply challenges, some participants affirmed encountering no challenges during EXIT-TB implementation. On the one hand, those who affirmed encountering no challenge were mainly those who performed coordination roles, and they linked this to the efficient delivery of materials and supplies from implementing partners. For example, a district TB and Leprosy coordinator in Tanzania affirmed that in case of a lack of X-ray films, NIMR was supplying them timely. On the other hand, the many who affirmed encountering equipment and supply challenges reported that most of them were eventually solved, or alternatives implemented at some point. These challenges included: inadequate supply of stool containers particularly in Tanzania; absence of X-ray machines in some facilities or dysfunctional X-ray machines in some facilities which necessitated referring clients to other facilities and stock-out of cartilages for GeneXpert machines. Problems with X-rays and GeneXpert functionality and inadequate materials related to these machines, particularly films and Cartilages respectively emerged as the dominant barriers across countries. A participant in Tanzania mentioned receiving expired X-ray films which could not generate X-ray images although these were then replaced. A participant in Kenya indicated not using the GeneXpert machine for the last two months because of the absence of cartridges necessitating the use of smear microscopy, which was perceived as less sensitive, less likely to detect drug resistance, requires two samples- spot and early morning samples and has long waiting time for the results (24–48 h). GeneXpert was perceived as highly sensitive, more likely to detect drug resistance detection, only requiring spot samples which reduces the burden of returning for early morning samples among clients and had short result processing time. There were also concerns about the absence of PPEs and delays in X-ray results. Delays in X-ray results were partly linked to limited expertise in the interpretation of the results, particularly in Ethiopia.

### Demand side challenges

Participants’ description of demand side challenges can be grouped into (i) negative clients’ behaviours; (ii) limited community awareness about TB and negative beliefs and fears; (iii) inability to meet financial demands of further care when needed. Each of these challenges are examined in detail next.

#### Negative clients’ behaviours’

Some participants mentioned negative client behaviours including delayed healthcare seeking among those with TB symptoms, coming late to the facilities, attending the facilities on the days in which TB screening is not performed (e.g., weekends) and/or not returning to the facility when they fail to produce sputum on the day they were screened or after initiation of treatment.

#### Limited community awareness about TB, negative beliefs and fears

Lack of awareness among community members of what TB is, its transmission and prevention, as well as treatment, emerged as common in the accounts of participants in all countries. Relatedly, some participants mentioned negative beliefs and fears among clients. There were beliefs that going for TB screening means having the disease and hesitancy among some people because of beliefs that people who are conducting TB screening are generating income from identifying TB clients. Furthermore, there were fears of the stigma associated with the isolation of those with TB symptoms (to minimize transmission) and fears that testing TB positive may expose them to stigma and discrimination from community members. Likewise, there were fears of screening for TB among some clients because some relate TB screening to HIV screening which contributed to the denial of having TB symptoms because of fear that they will also be screened for HIV. Some described fears of coming to the hospitals because of equating TB symptoms such as cough to COVID-19 during the pandemic, and refusal of putting on a face mask because of fear of stigma. Some however indicated that intensive health education and prioritisation of TB patients to receive faster care were used to offset these fears and beliefs although they persisted among some clients.

#### Financial challenges

Financial challenges among clients dominated participants’ descriptions. Common financial challenges included clients’ non-attendance to X-ray services because of fear of being charged money and failure to meet financial demand for conducting Chest X-rays in a referral facility from where initial screening was performed. There were also challenges of failure to meet the cost of X-ray films were required to pay (e.g., in Uganda) and long distances to facilities in the absence of adequate funds to meet the cost of travel. It is important to note that one participant in Ethiopia mentioned the provision of transport of patients to the nearest facility for X-ray services as a way of addressing financial challenges; however, such support was cited to end when the EXIT-TB project ended.

### Theme 3: Suggestions for scaling up the EXIT-TB package

The suggestions for scaling up the EXIT-TB package were fourfold. First, increasing human resources for instance TB screeners and providers. One clinical officer in Kenya suggested that ‘more screeners and staff are needed in facilities to facilitate scaling up. Second, ensuring effective communication between leaders and ground-level implementers. Effective communication of challenges between implementers who encounter these challenges on the ground and leaders who are responsible for addressing some of these challenges was emphasised in Ethiopia. Third, few participants recommended financial incentives to healthcare providers. EXIT-TB focal persons in Tanzania regarded financial incentives to providers as a motivation for them to ‘identify more TB cases’ because they are few compared to the workload. Fourth and final, training and capacity building of implementers. Some recommended training before implementation, refresher training and additional rounds of training particularly on X-ray implementation as this emerged problematic among some clinicians.

The dominant recommendations for improving access to equipment and supplies were related to X-ray services. First, recommendations to ensure physical access to X-ray services. Many participants in all countries recommended the Government ensures the availability of X-ray machines in all facilities to reduce the time and financial resources needed to go for X-ray services in facilities other than where initial screening was conducted. Second, recommendations were to ensure financial access to X-ray services. Many participants recommended the continuation of subsidised or free X-ray services. Note that free X-ray services emerged as one of the drivers of project success. This explains why most participants recommended subsiding or waiving the cost of X-ray services as part of scaling up of EXIT-TB package. One EXIT-TB focal person in Tanzania went ahead to recommend the expansion of basic insurance coverage to meet the cost of X-rays. This recommendation was made given the minimum benefits offered by the Community Health Fund (CHF)- an affordable insurance scheme in Tanzania that does not cover the cost of X-ray services. Third, ensuring the availability of human resources required for X-ray services i.e., radiologists. Fourth and final, there was a recommendation to ensure a sustainable supply of X-ray films. Related to this, was a recurrent recommendation of ensuring the availability of GeneXpert cartridges as well as other materials such as registers.

Some participants emphasised the need to improve infrastructure for TB services. Such recommendations were common in facilities where space for offering TB services was challenging. For instance, a participant in Kenya recommended redesigning rooms used for TB services to increase space. Relatedly, few participants suggested improved project financing through Government takeover particularly the Ministry of Health and implementing partners to help a wider scale-up. This suggestion was made in view that it is the Government’s responsibility to control TB transmission. The strategies for Government takeover suggested in Ethiopia included integration of this program into the existing health system, assigning personnel to this program without a need for payment apart from their salaries and use of existing resources. Related to this, some participants recommended an extension of implementation time for the project based on its notable success. Some participants commented:

*For the government to implement this at a large scale, that needs to ensure the availability of X-rays in all facilities as part of the package (CHW, Tanzania)*

*I will advocate for improving the supply of TB testing materials to ensure that the machines run with no interruptions. Emphasising continuous screening with no laboratory materials is a waste of effort. So, at least they need to ensure a regular supply of cartridges for GeneXpert for sustainability (Clinical Officer, Uganda)*

*It would be very good if this program was not interrupted… We should not wait to be funded externally again, it would be better if the government can assign personnel in this area by paying their salary and we health professionals continue implementing this program without inquiring about an extra payment. Also, we can use the government resources we already have and let the program continue. For example, there are two Ambulances here in our centre so we can use one in the countryside and one here in the city. The Government needs to implement this package. If the government incorporates this EXIT-TB program into its health system and implements it throughout the country, I believe TB can be eradicated like malaria from our country (Health Extension Nurse, Ethiopia)*

Fears and negative beliefs and behaviours emerged as challenges of the EXIT-TB project, necessitating the need to address stigma and fears associated with TB. One clinician in Kenya suggested that ‘there is a *stigma about TB and then there is that fear factor”* without offering any details. To address these fears and stigma, some participants suggested improving community awareness about TB diseases through community education and health talks. Few participants expressed concerns about loss to follow-up clients e.g., those who are not returning after failing to produce sputum on the day they were screened or those initiated on treatment (see above). There were also concerns about inadequate contact tracing that emerged among some participants (see above). Consequently, some participants recommended more use of link assistants for tracking lost clients within the communities and linking them to the facilities. There were also recommendations to increase CHW allowance and/or facilitate transport support for CHWs to conduct contact tracing. Some participants commented:

*The things that would contribute to success. Scaling up includes educating and giving health talks to communities. Training the community by emphasizing why they should get screened is important to reduce fears and stigma towards the disease (Community Volunteer, Uganda)*

*There is a need to maximise the engagement of link assistants because they assist in identifying people with coughs and link them to clinicians. Once the clinicians investigate and find positive patients, some of them get lost along the way. Therefore, link assistants can be useful in tracking and linking them to TB treatment services (Clinical Officer, Kenya)*

## Discussion

This paper examined the contribution of EXIT-TB, challenges encountered during implementation and suggestions for scaling up the EXIT-TB package in Tanzania, Kenya, Uganda, and Ethiopia. The implementation of the EXIT-TB package was done in recognition of low TB case detection in EA despite a decline in prevalence, incidence, and death rates globally [1, 17–22]. IDIs were conducted with service providers, policymakers, and other implementing partners with a focus on their experiences with EXIT-TB implementation, changes noticed and their insights on important considerations for scaling up the package in a similar or another setting.

The findings indicate a broad consensus that EXIT-TB has contributed to an increase in TB screening and diagnosis in the study settings. The findings further indicated an improvement in TB service delivery by reducing waiting time for screening and diagnosis. Healthcare providers appear to have benefited from EXIT-TB implementation by gaining more competence in conducting TB screening and diagnosis and improving their decision-making capacity. The provision of chest X-rays as screening and diagnostic tools and free X-ray services for those who could not afford them facilitated more TB case finding. Likewise, TB case finding integration to other clinics, community sensitization and cooperation between providers at the facility and community as well as better linkage of clients from communities to facilities emerged as major contributors to the success noted. One of the important practices emerging in our study was the engagement of CHWs in community sensitization and screening at the facility as a driver of increased screening. Another important finding was the use of new simple screening criteria as the key drivers of TB screening uptake. During EXIT-TB implementation, anyone presenting with a cough of any duration was screened contrary to the guideline in implementation countries where a cough of more than two weeks is often considered. Our findings strongly mirror the findings of most of the previous studies. Evidence continues to indicate that integrated TB screening packages have significantly increased TB diagnosis in many countries. For instance, a recent study in Ghana suggests that an integrated TB screening package with a simplified process for TB screening, linkage, integration of screening services across service delivery points and referral and engagement of community health care providers are the major drivers of increased TB screening [11, 23]. Another study in Eswatini indicated an increase in TB screening among pregnant women because of the integration of TB/HIV services in Reproductive Maternal Neonatal and Child Health settings despite some concerns with symptom screening [24]. Furthermore, it is important to note that the contribution of CHWs in driving the success of TB screening interventions has been widely documented [25-27]. In India for instance, CHW has been documented to be critical in the success of active TB case findings [25]. In Mozambique, CHWs have been the driving force behind the success of facility-based TB screening [26]. This indicates that the value of employing CHWs in integrated TB screening activities is indispensable. This explains why Sinha, Shenoi & Friedland [27], have illustrated the effectiveness of CHWs across the entire cascade of TB care and outlined additional opportunities for CHWs to address challenges particular to the TB pandemic. Taken together, these findings indicate that integration of TB screening into routine care, affordability of screening tests, use of CHWs for community sensitization and screening and use of simplified screening criteria are critical in increasing TB screening and diagnosis.

The study unmasked a range of supply and demand-side challenges related to the implementation of the EXIT-TB package and the entire TB program in the countries. On the one hand, key supply-side challenges included practice-and resource challenges. Important practice challenges included the concerns of disorganised care in some facilities and negative attitudes towards the project and TB-Diagnostic procedure among some HCWs. Relatedly, important resource challenges included infrastructure barriers and human resources for health issues in terms of expertise and quantity as well as an inadequate stock of essential materials. Dysfunctional or absence of X-Ray and GeneXpert services in some healthcare facilities emerged as a recurrent challenge. Looking across the literature, similar practice and resource challenges have been widely documented on the supply side as facing not only TB screening but also healthcare service provision as a whole. Specifically, to the implementation of integrated TB packages, practice challenges such as the negativity of HCWs and resource challenges such as infrastructure, equipment, essential materials, and human resource gaps have been documented to Impact TB screening in some African countries [2, 4, 5, 11, 14, 23, 28, 29]. For instance, a recent qualitative study examining factors that influence the implementation of TB screening among PLHIV in selected HIV clinics in Ghana [23] reported negative attitudes and low commitment of HCWs to TB screening and limited facility infrastructure as the main barriers. Consequently, the need to increase HCWs’ commitment towards TB screening interventions was recommended by the authors. Within East Africa, concerns of understaffing, inadequate diagnostic materials, service disorganization and malpractice have been recently identified by our team as impacting TB Diagnosis in Kenya, Uganda, and Tanzania [11]. This indicates that the success of TB Screening interventions requires addressing both practice and resource challenges in healthcare facilities.

On the other hand, the key demand side challenges emerging from our study included delayed care seeking, immature discontinuation of the screening process because of failure to return to the facility, negative beliefs, fears of stigma towards screening and financial challenges. Similar to supply-side challenges, demand-side challenges have been widely discussed in the literature. A common approach in most literature is to document both supply and demand sides concurrently. For example, studies in Uganda have documented concerns of infrastructure, understaffing and expertise concerns on the supply side and stigma and financial challenges on the demand side as the barriers to TB screening [28, 29]. However, a few works of literature have specifically highlighted the demand side barriers to TB screening. For example, a qualitative study of TB patients in Mozambique identified concerns of stigma related to diagnosis and treatment, inadequate knowledge, and negative beliefs as among the barriers to TB diagnosis and treatment [30]. The existence of these challenges may explain why participants suggested improvement of service delivery, access to diagnostic equipment and supplies, physical infrastructure, and financing, addressing client fears and stigma, and improving the linkage of clients from communities to facilities for scaling up of EXIT-TB packages. This indicates that the success of the integrated TB screening package largely depends on the efforts to address both the supply and demand side challenges more broadly.

### Limitations

While the implementation of the EXIT-TB package in multiple countries is a major strength, this may also be a limitation. The implementation of the EXIT-TB package across many countries with many healthcare facilities covering rural and urban settings. While a focus on fewer countries could have resulted in richer data, we believe that multi-country studies are important in generating evidence that can be easily adopted globally. Some researchers have provided evidence to support this notion [31].

## Conclusion

The findings of this study indicated that the EXIT-TB intervention was described to have facilitated an increase in TB case detection and reduced delay in Tanzania, Kenya, Uganda, and Ethiopia. However, similar to other public health programs, the implementation of the EXIT-TB package is not without challenges. Both supply and demand side challenges were mentioned indicating that addressing these challenges is necessary to maximise the success of the EXIT-TB package. More specifically, addressing the challenges of service delivery, infrastructure, equipment and supplies as well as demand side challenges of TB knowledge, screening fears and linkage is needed to maximise the impact of the EXIT-TB package.

## Supplementary Information

**Additional file 1.** National institute for medical research - Muhimbili EXIT-TB Project.

## Data Availability

The datasets used and/or analyzed during the current study are available from the corresponding author on reasonable request.

## References

[1] World Health Organization [WHO]. Global Tuberculosis Report 2021. Geneva; 2021. https://www.who.int/publications-detail-redirect/9789240037021.

[2] Mohammed H, Oljira L, Roba KT, Ngadaya E, Manyazewal T, Ajeme T, Mnyambwa NP, Fekadu A, Yimer G (2022). Tuberculosis prevalence and predictors among health care-seeking people screened for cough of any duration in Ethiopia: a multicenter cross-sectional study. Front Public Health.

[3] MoHCDGEC. The United Republic of Tanzania, The National Tuberculosis and Leprosy Programme Annual Report 2019. Ministry Health Community Development Gender, Elderly and Children; 2019.

[4] Uganda National Tuberculosis and Leprosy Control Programme. Manual for management and control of Tuberculosis and Leprosy in Uganda. 2017;(3rd edition):1–177.

[5] Eliso E, Medhin G, Belay M (2015). Prevalence of smear-positive pulmonary tuberculosis among outpatients presenting with cough of any duration in Shashogo Woreda, Southern Ethiopia. BMC Public Health.

[6] NTLP. Manual for Management of Tuberculosis and Leprosy in Tanzania. 2019;285–7.

[7] Dujardin B, Kegels G, Buvé A, Mercenier P (1997). Tuberculosis control: did the programme fail or did we fail the programme?. Trop Med Int Health.

[8] WHO. Chest Radiography in Tuberculosis. World Health Organization, Geneva. 2016;1–44.

[9] WHO. SURVEYS: handbook. Tuberculosis. World Health Organization, Geneva; 2011.

[10] World Health Organization [WHO]. Xpert MTB/RIF implementation manual; Technical and operational “how-to”: practical considerations. World Health Organization. Geneva; 2014. 25473699

[11] Mnyambwa NP, Philbert D, Kimaro G (2021). Gaps related to screening and diagnosis of tuberculosis in care cascade in selected health facilities in East Africa countries: a retrospective study. J Clin Tuberc Other Mycobact Dis.

[12] Bradshaw C, Atkinson S, Doody O (2017). Employing a qualitative description approach in health care research. Glob Qual Nurs Res.

[13] National Tuberculosis Leprosy and Lung Disease Program. Annual Report 2021. Ministry of Health, Kenya; 2021.

[14] Alene KA, Clements ACA (2019). Spatial clustering of notified Tuberculosis in Ethiopia: a nationawide study. PLoS ONE.

[15] Baluku JB, Nanyonjo R, Ayo J, Obwalatum JE, Nakaweesi J, Senyimba C, et al. Trends of notification rates and treatment outcomes of tuberculosis cases with and without HIV co-infection in eight rural districts of Uganda ( 2015–2019 ). BMC Public Health [Internet]. 2022;1–10. 10.1186/s12889-022-13111-1 PMC8981742 35382794

[16] Braun V, Clarke V (2006). Qualitative Research in Psychology Using thematic analysis in psychology Using thematic analysis in psychology. Qual Res Psychol [Internet].

[17] Welday SH (2014). Gene Xpert testing of stool samples for the diagnosis of pulmonary tuberculosis in children less than 15 years in hospitals in and around Nairobi. Int J Tuberc Lung Dis.

[18] Muttamba W, Tumwebaze R, Mugenyi L, Batte C, Sekibira R, Nkolo A (2020). Households experiencing catastrophic costs due to tuberculosis in Uganda: magnitude and cost drivers. BMC Public Health.

[19] Dangisso MH, Datiko DG, Lindtjørn B (2015). Low case notification rates of childhood tuberculosis in southern Ethiopia. BMC Pediatr.

[20] WHO. Tuberculosis Fact Sheet. World Health Organisation, Geneva; 2020.

[21] WHO. High Burden Countries for Tuberculosis | Stop TB Partnership. World Health Organization, Geneva; 2019.

[22] WHO. SDG Target 3 Communicable diseases. World Health Organization; Geneva;2019.

[23] Narh-Bana SA, Kawonga M, Odopey SA, Bonsu F, Ibisomi L, Chirwa TF (2022). Factors influencing the implementation of TB screening among PLHIV in selected HIV clinics in Ghana: a qualitative study. BMC Health Serv Res.

[24] Hartsough K, Teasdale CA, Shongwe S, Geller A, Pimentel De Gusmao E, Dlamini P (2022). Enhanced integration of TB services in reproductive maternal newborn and child health (RMNCH) settings in Eswatini. PLOS Glob Public Health..

[25] Garg T, Bhardwaj M, Deo S (2020). Role of community health workers in improving cost efficiency in an active case finding tuberculosis programme: an operational research study from rural Bihar, India. BMJ Open.

[26] José B, Manhiça I, Jones J, Mutaquiha C, Zindoga P, Eduardo I (2020). Using community health workers for facility and community-based TB case finding: an evaluation in central Mozambique. PLoS ONE.

[27] Sinha P, Shenoi SV, Friedland GH (2020). Opportunities for community health workers to contribute to global efforts to end tuberculosis. Glob Public Health.

[28] Nansera D, Bajunirwe F, Kabakyenga J, Asiimwe PKJ, Mayanja-Kizza H (2010). Opportunities and barriers for implementation of integrated TB and HIV care in lower level health units: experiences from a rural western Ugandan district. Afr Health Sci.

[29] Ayakaka I, Ackerman S, Ggita JM, Kajubi P, Dowdy D, Haberer JE (2017). Identifying barriers to and facilitators of tuberculosis contact investigation in Kampala, Uganda: a behavioral approach. Implement Sci.

[30] De Schacht C, Mutaquiha C, Faria F (2019). Barriers to access and adherence to tuberculosis services, as perceived by patients: a qualitative study in Mozambique. PLoS ONE.

[31] Daviaud E, Owen H, Pitt C, Kerber K, Bianchi Jassir F, Barger D (2017). Overview, methods and results of multi-country community-based maternal and newborn care economic analysis. Health Policy Plan.

