# Supplementary material for: Implementation of evidence-based multiple focus integrated intensified TB screening to end TB (EXIT-TB) package in East Africa: a qualitative study
Source: BMC Infect Dis. 2023 Mar 14;23:161. doi: 10.1186/s12879-023-08069-3 (PMC10013287; doi:10.1186/s12879-023-08069-3)
Supplement: Supplementary file 1 — Additional file 1. National institute for medical research - Muhimbili EXIT-TB Project. [file 12879_2023_8069_MOESM1_ESM.docx]

**NATIONAL INSTITUTE FOR MEDICAL RESEARCH - MUHIMBILI**

**EXIT-TB PROJECT**

This is a short questions’ questionnaire aiming to understand **Feasibility**, **Barriers** and **Challenges** during the implementation of EXIT-TB.

Interviewees:

- EXIT-TB Focal Person
- Medical Officer In-charge (Facility In-charge)
- TB Focal Person/Clinician involved in EXIT-TB activities
- Community Health Worker/Volunteer involved in EXIT-TB Project

Questions:

1. How did the health care providers facilitate the implementation of EXIT-TB package?
2. Screening all patients for TB who **passively** report cough at OPD and RCH clinics
3. Active screening of all children with a contact with TB
4. Testing for TB irrespective of TB symptoms among all patients with advanced HIV/AIDS diseases (*CD4 < 200* cells/mm^3^ and/or WHO stage 3 or 4)
5. Actively screen for TB among diabetic patients
6. What were the physical barriers (stock-out of supplies (x-ray films, lab supplies)) in scaling-up EXIT-TB package?
7. What are the technical capacity barriers (trainings, technical capacity of reading x-rays, use of equipment (x-rays, GeneXpert, computers)) in implementation of the EXIT-TB package?
8. What are the provider-related barriers (staff overload, low staff motivation, and poor coordination of health facilities, leadership) in implementation of the EXIT-TB package in urban and rural settings?
9. What are the challenges faced by health care providers in delivering EXIT-TB package?
10. What change has EXIT-TB brought in your facility even after it was phased out?
11. If the government wish to scale up the EXIT-TB package, what would you recommend?
